# Expanded Radiologic Characterization and Long-Term Imaging Follow-Up of Sclerosing Angiomatoid Nodular Transformation (SANT): A Case Report

**DOI:** 10.7759/cureus.102105

**Published:** 2026-01-22

**Authors:** Usama Arbab, Rajoo Ramachandran, Sowmya Gopalan, P.M. Venkata Sai

**Affiliations:** 1 Department of Radiology, Sri Ramachandra Institute of Higher Education and Research, Chennai, IND; 2 Department of General Medicine, Sri Ramachandra Institute of Higher Education and Research, Chennai, IND

**Keywords:** acute hepatitis b, benign lesion, rare splenic neoplasm, red pulp, sclerosing angiomatoid nodular transformation (sant), solitary lesion, splenic vascular lesion

## Abstract

Sclerosing angiomatoid nodular transformation (SANT) is an uncommon, non-malignant vascular lesion of the spleen that can resemble malignant lesions during imaging studies. Histologically, the spleen comprises two parts: the red pulp, which acts as a blood filter, and the white pulp, which plays a crucial role in immunity. Primary splenic neoplasms are classified into lymphoid neoplasms, which arise from the white pulp, and vascular neoplasms, which arise from the red pulp. Lesions arising from vascular elements include benign lesions, such as hemangioma, hamartoma, and SANT, as well as intermediate or variable lesions, including hemangioendothelioma, hemangiopericytoma, and littoral cell angioma. Lastly, there are malignant lesions such as angiosarcoma. This report presents the case of a patient with a solitary, well-defined splenic mass on abdominal imaging. The patient had recently been diagnosed with acute hepatitis B and was in the cholestatic phase at the time the splenic lesion was identified. He had been initiated on tenofovir therapy. The lesion demonstrated characteristic radiological features of SANT. Histopathological examination confirmed the diagnosis of SANT. This case highlights the importance of considering SANT in the differential diagnosis of splenic masses, particularly when imaging findings are inconclusive. Early recognition and appropriate management can prevent unnecessary invasive procedures and psychological trauma. This report provides an expanded radiologic perspective and long-term imaging follow-up of a case previously described from a clinical viewpoint.

## Introduction

Sclerosing angiomatoid nodular transformation (SANT) of the spleen is an uncommon, non-malignant vascular lesion initially characterized and reported by Martel et al. in 2004 [1]. It originates from the red pulp of the spleen and is characterized by multiple angiomatoid nodules surrounded by dense fibrotic stroma [1]. SANT is typically asymptomatic and is often found incidentally during imaging for unrelated conditions [1]. Radiologically, it shows distinct features, including a hypovascular center with peripheral and septal enhancement, frequently described as a "spoke-wheel" pattern on contrast-enhanced studies [2]. Determining the exact prevalence of SANT is challenging due to its broad differential diagnosis. Other splenic lesions, such as hemangioma, hamartoma, lymphoma, and metastases, can mimic its appearance, making histopathological examination necessary for definitive diagnosis [2]. This patient had previously been reported in a medicine-focused case report that primarily described the clinical and hepatologic features [3]. However, detailed radiologic analysis, advanced magnetic resonance imaging (MRI) characterization, and extended imaging follow-up were not addressed in that report. The present case re-evaluates the same patient from a radiologic-pathologic perspective, emphasizing imaging findings and long-term evolution.

## Case presentation

A 41-year-old man, recently diagnosed with acute hepatitis B and in the cholestatic stage at the time of presentation, was undergoing treatment with tenofovir. He presented with a three-day history of fever, abdominal pain, and vomiting. Laboratory investigations revealed elevated total count and liver function test values (Table 1).

**Table 1:** Laboratory findings SGOT, serum glutamic-oxaloacetic transaminase; SGPT, serum glutamic-pyruvic transaminase; WBC, white blood cell

Parameter	Value	Reference
Hemoglobin	15.3 g/dL	13-17 g/dL
Total WBC count	12,400 cells/mm³	4,000-11,000 cells/mm³
Polymorphs	80.2%	45-70%
SGOT	831 U/L	<40 U/L
SGPT	1,246 U/L	<41 U/L
Total bilirubin	2.01 mg/dL	<1.2 mg/dL
Direct bilirubin	0.62 mg/dL	<0.2 mg/dL
Indirect bilirubin	1.39 mg/dL	0.1-1.0 mg/dL

**Figure 1:**
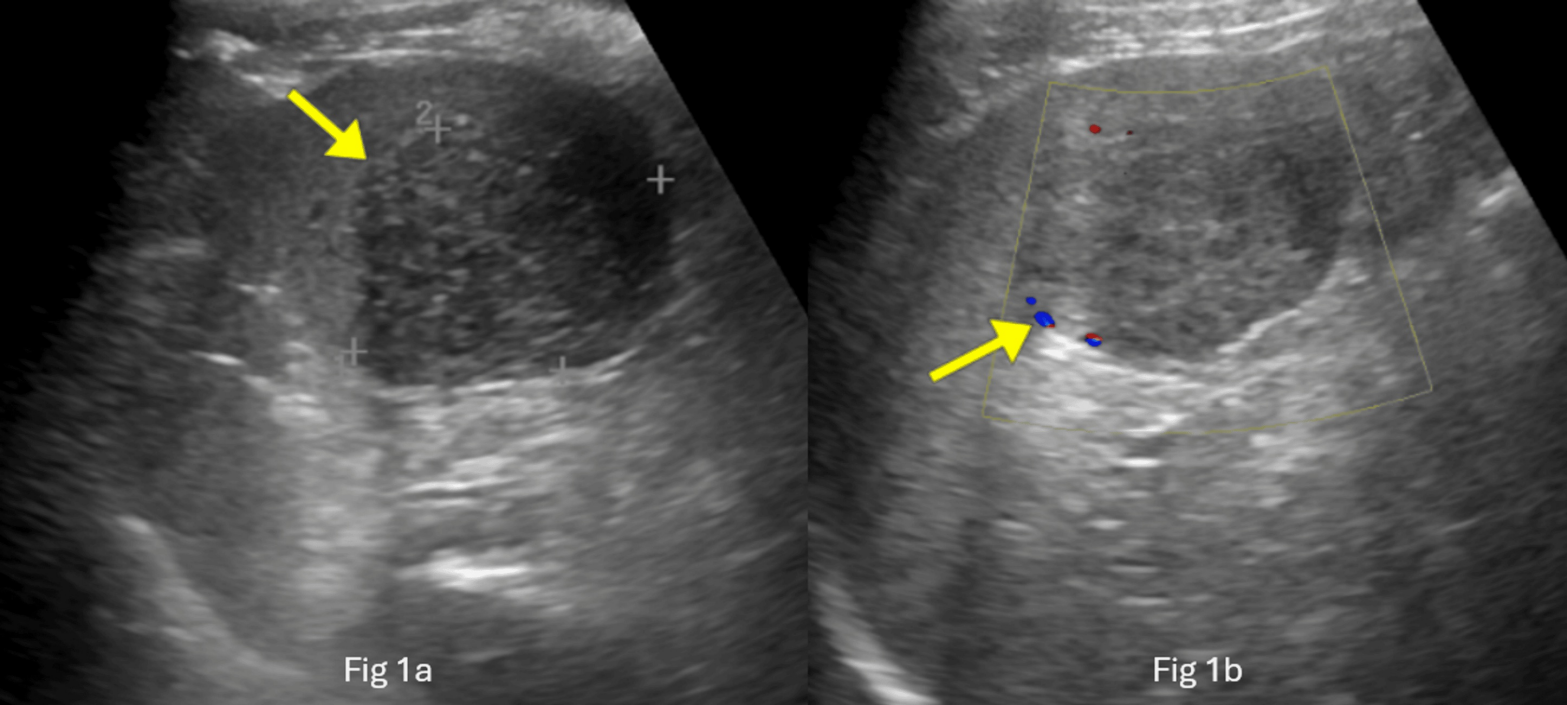
Ultrasound of the abdomen A large, well-circumscribed, heterogeneous lesion measuring 5.0 x 3.4 cm indicated by the yellow arrow in the lower pole of the spleen (1a), with only minimal peripheral flow on color Doppler (1b).

An abdominal ultrasound (USG) revealed a normal-sized spleen (9.0 cm) with a large, well-circumscribed, heterogeneous mass/lesion measuring 5.0 x 3.4 cm in the lower pole of the spleen, with only minimal peripheral flow on color doppler, raising concern for a splenic abscess (Figures 1a, 1b).

**Figure 2:**
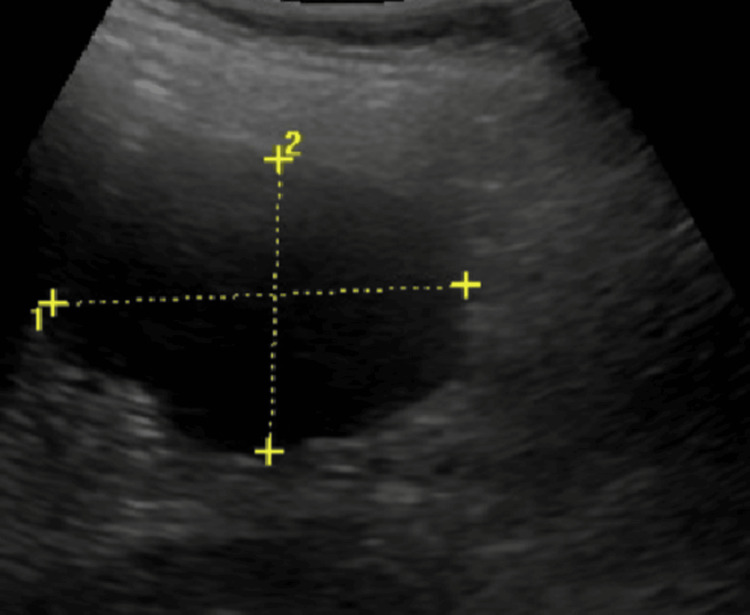
Repeat ultrasound of the abdomen The five-day follow-up ultrasound showed no significant interval change (5.0 × 3.5 cm).

The patient was started on vancomycin 1 gram twice a day and ceftriaxone 1 gram twice a day. A follow-up USG was performed after five days, which showed no interval change (size 5.0 x 3.5 cm) (Figure 2).

**Figure 3:**
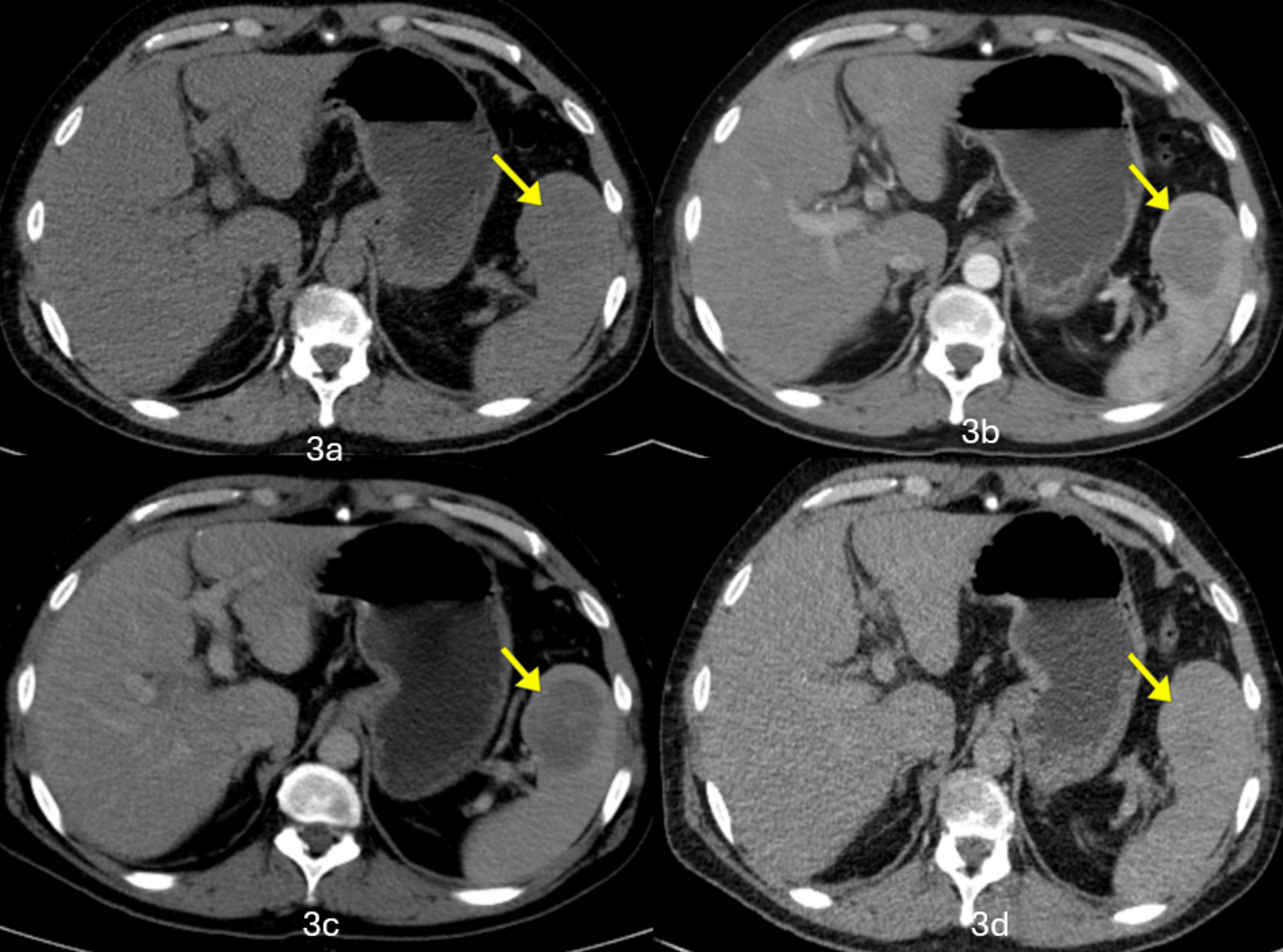
CECT of the abdomen CECT showing a well-defined hypodense lesion in lower pole of the spleen, measuring 4.8 x 3.3 cm (yellow arrow) (3a) with peripheral arterial enhancement (3b) and progressive centripetal filling in the venous (3c) and delayed (3d) images, producing a characteristic "spoke-wheel" pattern of enhancement. CECT, contrast-enhanced computed tomography

Contrast-enhanced computed tomography (CECT) was performed, which revealed a well-defined hypodense lesion, measuring 4.8 x 3.3 cm, showing peripheral arterial enhancement and progressive centripetal filling in the venous delayed images in the lower pole of the spleen (Figure 3), which raised the suspicion of a primary vascular tumor, and an MRI of the abdomen was suggested.

**Figure 4:**
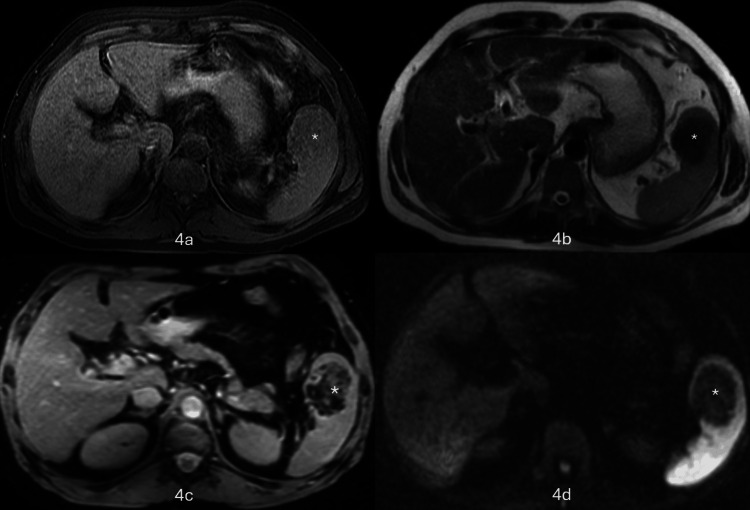
MRI of the abdomen MRI showing a T1-isointense lesion (star) with respect to the spleen (4a) and a T2-heterogeneously hypointense lesion (4b). Susceptibility artefact on GRE sequence (star) is suggestive of hemosiderin deposition within the lesion (4c). There is no evidence of diffusion restriction (star) on diffusion-weighted imaging (DWI) sequence (4d).

An MRI of the abdomen was performed, which showed a T1-isointense lesion measuring 5.0 x 3.4 cm with respect to the spleen and a T2-heterogeneously hypointense lesion with susceptibility artefact on GRE, suggestive of hemosiderin deposition within the lesion (Figures 4a-4c). However, no evidence of diffusion restriction was noted (Figure 4d).

**Figure 5:**
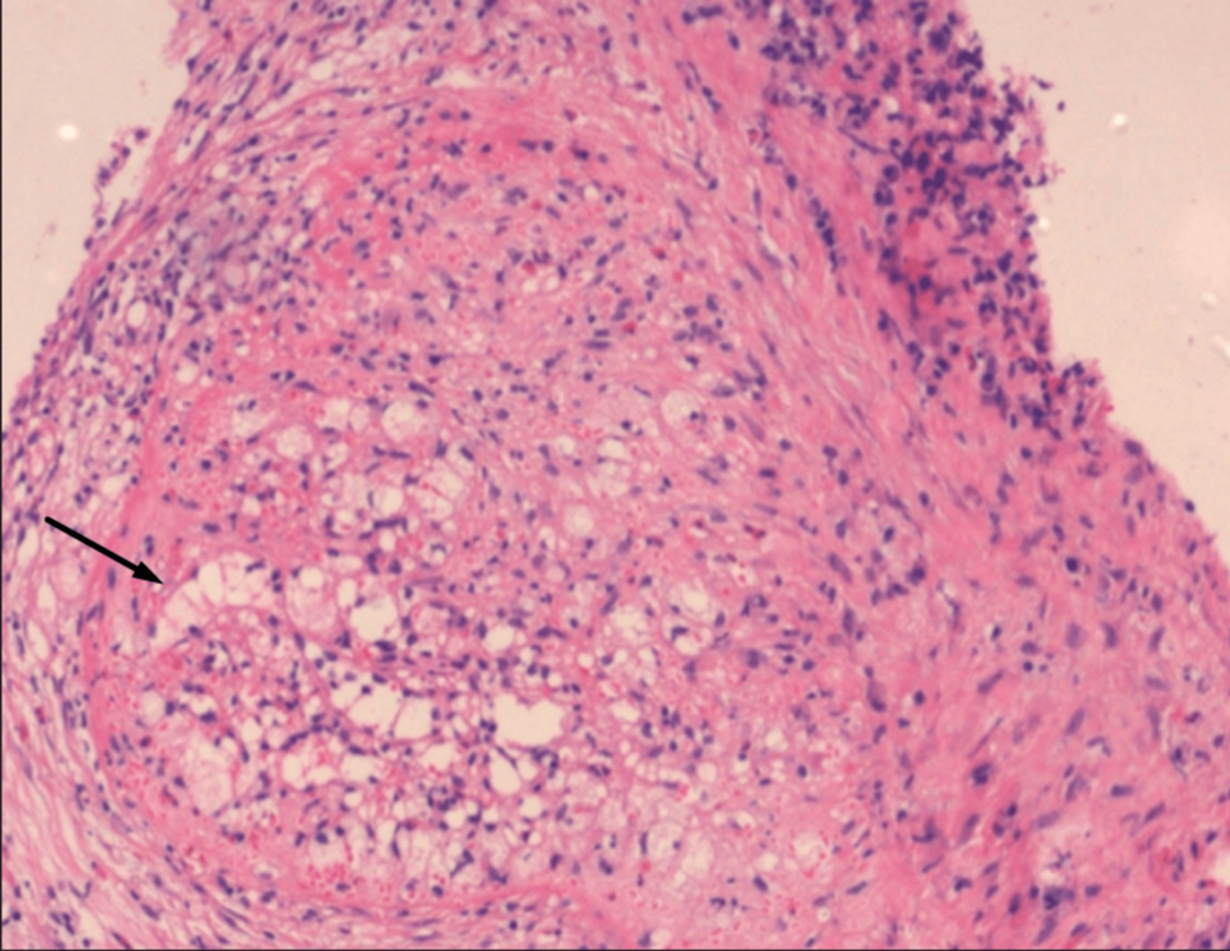
Histopathological examination Disorganized blood vessels arranged as nodules on hematoxylin and eosin (H&E) staining (black arrow).

A USG-guided Tru-cut biopsy was performed, which showed disorganized blood vessels arranged as nodules (Figure 5).

**Figure 6:**
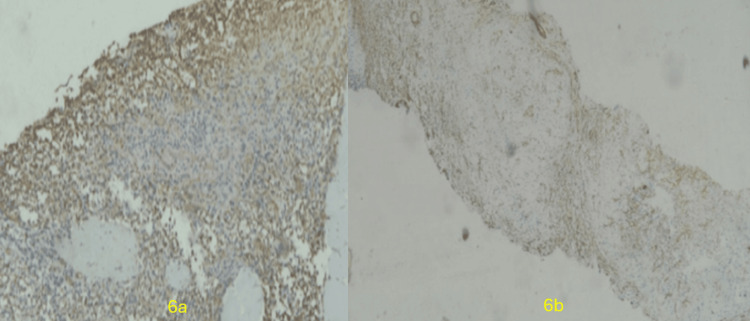
Immunohistochemical staining Immunohistochemistry showing vascular positivity for CD34 (6a) and SMA (6b).

The lesion demonstrated vascular positivity for CD34 and SMA on immunohistochemistry (Figure 6), findings that favor the diagnosis of SANT of the spleen.

The patient was counselled regarding the benign nature of the lesion and the need for surveillance. He understood and agreed with conservative management, remained compliant with follow-up visits, and reported no concerns during the surveillance period. At three months, repeat USG revealed no significant change in lesion size (4.5 x 3.7 cm).

**Figure 7:**
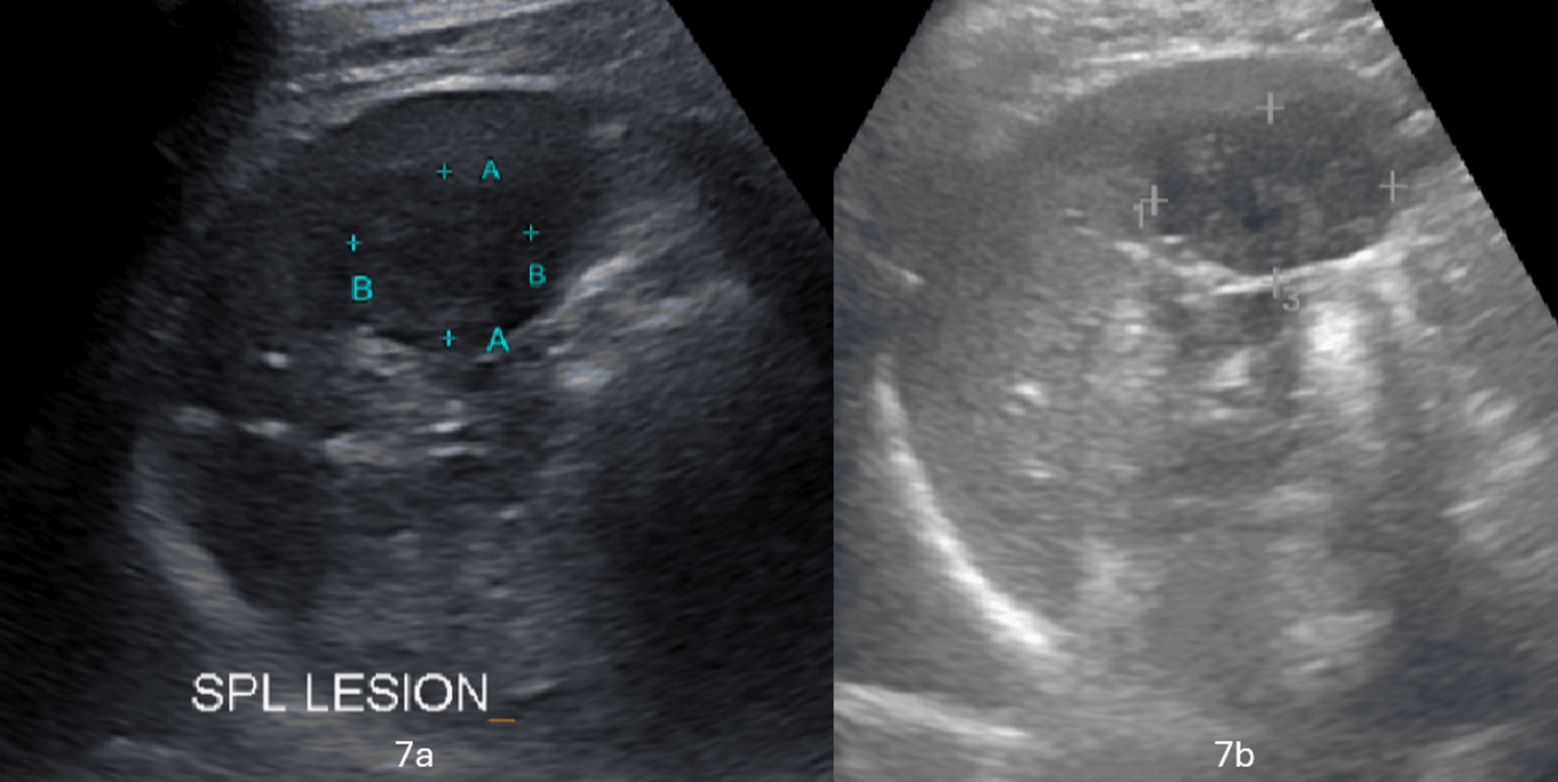
Follow-up ultrasound Ten-month (7a) and two-year (7b) follow-up images showing no significant interval change, measuring 4.5 × 4.3 cm and 4.3 × 4.0 cm, respectively.

Further follow-up scans at 10 months and 2 years demonstrated no appreciable change in lesion morphology, measuring 4.5 × 4.3 cm and 4.3 × 4.0 cm, respectively (Figures 7a, 7b, respectively).

## Discussion

SANT of the spleen is an uncommon, non-malignant vascular lesion. The term was first coined in 2004 [1]. The pathogenesis is unclear, but the histopathologic features suggest altered red pulp entrapment by exaggerated stromal proliferation/ augmentation due to inflammation, trauma, or hemorrhage [2]. It is usually an incidental finding in an otherwise asymptomatic individual. The underlying pathogenesis is still unclear. Martel et al. observed that the internodular stroma in most SANTs is similar to that in inflammatory pseudotumor (IPT) and proposed that SANT might be the final stage or a common endpoint for various splenic lesions, including IPT [1]. Similar to IPT, SANT has been linked to extrasplenic malignancies [4]. Weinreb et al. suggested that some cases of SANT may be related to an IPT of the spleen [5]. Rarely, patients can present with abdominal pain, anemia, and splenomegaly.

Radiologically, SANT appears solitary and heterogeneous on USG, with a greatest dimension of approximately 3-7 cm [6]. In contrast-enhanced USG (CEUS), a typical “Spoke wheel” pattern is seen [7]. Computed tomography (CT) shows early, peripheral rim enhancement with progressive centripetal “spoke wheel” pattern of enhancement [2]. Though CEUS was not performed on our patient, it is discussed here for contextual comparison. On MRI, the lesions are isointense on T1-weighted imaging [8], and there are radiating T2 hypointense bands extending towards the center, representing fibrous stroma [2]. Susceptibility artefacts indicate the presence of hemosiderin [2]. Diffusion-weighted imaging (DWI) exhibited a greater susceptibility effect compared to T2-weighted imaging and chemical shift imaging, leading to a more pronounced signal reduction. As a result, peripheral clear, multiple nodular isointense regions on DWI suggested angiomatoid nodules, while surrounding hypointense areas indicated fibrous tissue. Thus, DWI may be more effective than enhanced CT images and other MRI sequences for diagnosing SANT [9]. On positron emission tomography-computed tomography (PET-CT), fluorodeoxyglucose (FDG) uptake within the tumor is low and heterogeneous, consistent with prior reports and similar to inflammatory myofibroblastic lesions. While the degree of FDG accumulation may aid in distinguishing SANT from malignant splenic tumors, which typically have high FDG uptake, FDG-PET/CT alone cannot reliably differentiate SANT from other benign splenic lesions [10]. The observed F-18 FDG accumulation in SANT lesions indicates increased metabolic and glycolytic activity compared to normal splenic tissue, but this does not clarify whether SANT is a reactive or neoplastic process, as enhanced tissue metabolism may occur in both scenarios. Though PET-CT was not performed on our patient, it is discussed here for contextual comparison. Although SANT has been described in various clinical settings, detailed radiologic evaluation of SANT occurring in the setting of acute hepatitis B, particularly during the cholestatic phase, has not been previously elaborated. A previous report from our institution [3] described this same patient, with an emphasis on clinical and hepatologic features. The present report, however, provides expanded radiologic characterization, detailed MRI sequence analysis, and a two-year imaging follow-up not included in that publication.

Histopathologically, as described by Martel et al., the lesion displayed a multinodular architecture on low-power microscopy [1]. Each nodule demonstrated an angiomatoid pattern composed of slit-like, round, or irregular vascular spaces lined by plump endothelial cells interspersed with spindled or ovoid cells [1]. Several nodules, especially the smaller ones, were surrounded by concentric collagen rings. Numerous red blood cells and scattered inflammatory cells were present. Nuclear atypia was minimal, mitotic figures were exceedingly rare, and necrosis was absent. The inter-nodular stroma ranged from myxoid to densely fibrotic and contained scattered plump myofibroblasts, plasma cells, lymphocytes, and siderophages [1]. Immunohistochemistry revealed three distinct vascular components within the angiomatoid nodules: CD34+/CD8−/CD31+ capillaries, CD34−/CD8+/CD31+ sinusoids, and CD34−/CD8−/CD31+ small veins, resembling the normal vascular elements of the splenic red pulp [1].

Most reported cases of SANT have been managed with splenectomy; however, selected patients may be suitable for conservative “wait-and-watch” management with interval imaging surveillance [5]. Splenectomy carries an inherent risk of intraoperative and postoperative bleeding. To the best of our knowledge, the risk of rupture associated with SANT has not been documented. Furthermore, both the potential for rupture and the possibility of malignant transformation remain uncertain. To date, no instances of SANT recurrence have been reported. Since SANT is frequently identified incidentally, most patients remain asymptomatic, raising the question of whether routine observation could serve as a viable alternative to surgery. However, splenectomy carries notable risks, with reported morbidity rates around 30% and mortality rates reaching up to 15%, depending on the patient’s condition and surgical indication [11,12]. The procedure poses a significant risk of intraoperative and postoperative hemorrhage [12], and its major long-term complication is overwhelming post-splenectomy infection (OPSI), often caused by encapsulated organisms such as *Streptococcus pneumoniae* [13].

## Conclusions

SANT of the spleen is an uncommon, non-malignant vascular lesion of the spleen that can closely mimic malignant neoplasms on imaging. Recognizing its characteristic radiologic appearance, particularly the classic “spoke-wheel” enhancement pattern, helps refine the differential diagnosis and may prevent unnecessary splenectomy. Histopathological and immunohistochemical evaluation remain essential for establishing a definitive diagnosis. Although its exact etiology is uncertain, the occurrence of SANT in the setting of acute hepatitis B, as in the present case, highlights a rare but noteworthy association. Given its indolent behavior and the lack of documented recurrence or malignant transformation, conservative management with periodic imaging follow-up is appropriate in selected asymptomatic patients. Greater awareness of SANT among radiologists and clinicians is vital for accurate identification, informed patient counselling, and avoidance of overtreatment. This case also supplements previously published clinical data by providing detailed radiologic evaluation and long-term imaging stability. Radiology-focused case re-evaluation can offer additional diagnostic clarity and enhance understanding of rare splenic lesions such as SANT.
